# Factors influencing vocational college students’ creativity in online learning during the COVID-19 pandemic: The group comparison between male and female

**DOI:** 10.3389/fpsyg.2022.967890

**Published:** 2022-08-11

**Authors:** Xinchen Niu, Xueshi Wu

**Affiliations:** School of Education, Jiangxi Science and Technology Normal University, Nanchang, China

**Keywords:** online learning, creativity, technology acceptance model, expectation confirmation model, technology task fit model, gender

## Abstract

The COVID-19 pandemic has forced higher education institutions to shift their teaching activities from traditional face-to-face to online learning. This brings a great challenge to the creativity training of vocational college students, who not only learn theoretical knowledge but also cultivate technical skills. Therefore, it is very important to explore the influencing factors of online learning on students’ creativity during the epidemic. By relying on the related literature review, an extensive model is developed by integrating the expectation confirmation model (ECM), technology task fit model (TTF), and the technology acceptance model (TAM) to illustrate key factors that influence creativity. Based on the proposed model, theory-based hypotheses are tested through structural equation modeling employing empirical data gathered through a survey questionnaire of 229 students from different vocational colleges in China. In addition, to extend the analysis results, this paper performs a comparative analysis based on gender. The findings demonstrate that perceived ease of use and perceived usefulness significantly influence knowledge sharing; knowledge sharing significantly affects creativity. However, there is no substantial relationship between perceived usefulness and attitude, and no relationship between attitude and knowledge sharing. Moreover, a multi-group comparison shows that there is a significant gender difference between perceived ease of use and attitude. Based on the findings, theoretical and practical implications are discussed.

## Introduction

COVID-19 is an unprecedented crisis that has transformed countries around the world in just a few months, with the disease now spreading to more than 220 countries and territories (Mani et al., 2020). To effectively deal with the spread of the virus, countries began to implement quarantine policies. For many industries, the consequences of the pandemic are unstoppable and uncontrollable. Perhaps, the most significant of these are major changes in national education systems, so teachers and students have to adapt to new teaching methods, even though most may not be fully prepared to deal with the sudden changes (Crawford and Cifuentes-Faura, 2022). According to UNESCO, the closure of schools has led to 50% of students receiving education through the Internet. Many educational institutions maintain the continuity of education through online teaching to ensure that students can study normally (Alfadda and Mahdi, 2021). The demand for online learning during the epidemic is very large and growing rapidly (Moser et al., 2020). The online education market is expected to grow at an annual rate of 16.4% from 2016 to 2023. With the rapid development of the Internet, perhaps, the teaching mode of school will change in 10 to 15 years. Online learning is more and more favored by learners (Shahzad et al., 2020).

Creativity is defined as a person’s ability to generate new and useful ideas and come up with solutions to problems by recombining and matching information and knowledge (Amabile and Pratt, 2016). Training students’ creativity has become a critical task in a modern society that values innovation and creation (Wang and Miao, 2015). According to Jahnke et al. (2015), students’ creativity is becoming more important in the 21st century, and students with creativity are more curious and capable of coming up with novel ideas and problem-solving solutions. Simultaneously, the COVID-19 outbreak has resulted in an exponential increase in the use of online learning. Therefore, the research on students’ creativity in the online learning environment has attracted a large number of scholars’ close attention (Cohen and Davidovitch, 2020). For example, Afari et al. (2013) believe that all creativity begins with interaction, knowledge sharing, and motivation, so it is productive to cultivate creativity in an online learning environment. The study by García-García et al. (2017) demonstrates that online learning provides an open environment for students, enabling them to exchange knowledge with others and enhance their creativity. Zulfikar (2020) confirms that collaborative digital platforms have a significant impact on students’ creativity, initiative, and critical thinking. Therefore, it is possible to conclude that students’ use of online learning systems is critical to improving their creativity.

Previous research has shown that online learning has an impact on student creativity (Afari et al., 2013; García-García et al., 2017). For example, Lee (2018) used social technology and social capital theory to examine for the first time how knowledge sharing affects individual creativity among college students in online learning, as well as the mediating effect of knowledge sharing on individual creativity. Yeh et al. (2012) used the hybrid knowledge management model, to integrate online learning and the three core processes of knowledge management (knowledge sharing, knowledge internalization, and knowledge creation) to study how to improve students’ creativity. The results showed that the hybrid knowledge management model is effective in improving knowledge, dispositions, and creativity. The research of Rasheed et al. (2020) demonstrated that the use of social media is related to students’ creativity and participation. Knowledge sharing is the potential reason to explain these relationships. Tsai et al. (2015) investigated 579 students in the department of tourism and hotel management to study how positive and negative online learning environments are related to students’ motivation and knowledge-sharing behaviors, thus affecting creativity. The results revealed that a positive online learning environment is related to intrinsic motivation and knowledge sharing, thus promoting creativity. A negative online learning environment is not conducive to creativity.

However, few studies involved the influencing factors of vocational college students’ creativity in the online learning situation during COVID-19. Vocational education is crucial to the development of a country, promoting employment and enhancing competitiveness. Its unique value stems from its potential to improve learners’ ability to acquire knowledge, cultivate skills, and develop creativity (Teclehaimanot et al., 2012). Among them, knowledge acquisition and skill cultivation strive to improve the overall quality of learners, such as creative problem solving. In addition, the rapid development of information technology has led to disruptive changes in work, study, and life patterns, which makes it impossible for learners to master all the knowledge and skills to deal with the future world (Phan, 2021). Toivanen et al. (2011) pointed out that developing creativity is important in a highly competitive and changing learning environment, where students have to deal with market demands and keep their competitive edge.

Different from previous studies, this study aims to explore the influencing factors of vocational college students’ creativity in the online learning situation during COVID-19. The specific differences are as follows: Firstly, this study integrates the three models of TTF, ECM, and TAM as the theoretical basis, while previous studies on creativity are mainly from the perspective of social technology and social capital theory (Lee, 2018; Fatemi et al., 2022). Secondly, the research object is vocational college students. Thirdly, the multi-group analysis is conducted to test whether gender differences existed in the creativity of vocational college students. The findings may provide a new direction for the development of online learning after the epidemic. Therefore, the questions of this study are as follows:

RQ 1: What are the influencing factors of vocational college students’ creativity in online learning situation?

RQ 2: To what extent do the influencing factors explain the creativity of vocational college students?

RQ 3: Are there gender differences in the impact of influencing factors on the creativity of vocational college students?

## Research model, literature review, and hypotheses

### Research model

The TAM, TTF, and ECM models have been widely employed in various fields (Tam and Oliveira, 2016; Lin et al., 2017; Al-Adwan et al., 2021). However, with the deepening of research, TTF and TAM models have their own limitations. For example, Nance and Straub (1996) pointed out that TAM focused on the individual beliefs of technology acceptance from the perspective of perceived usefulness and perceived ease of use, that is, the user’s attitude toward information technology, but it lacked consideration of user differences and could not explain whether the technology required by the task could be provided. TTF, on the other hand, focuses on user acceptability of technology by considering the fit between task and technological qualities and pays relatively greater attention to object study (Dishaw and Strong, 1999; Strong et al., 2006; Widagdo and Susanto, 2016). While TTF includes tasks and technological characteristics, it does not incorporate the users’ accepted beliefs about technology, which are at the heart of TAM. The TAM and TTF models explain the use of information systems from different perspectives on tasks, technology, and individual users.

**Figure 1:**
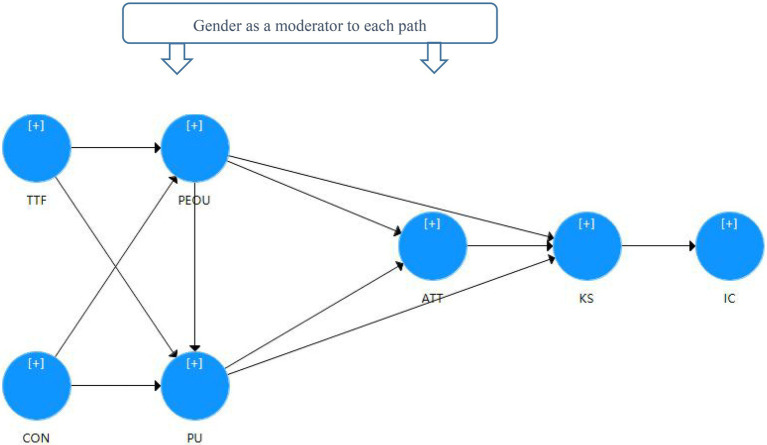
Research framework for online learning and creativity.

In order to improve the predictive relevance and coefficient of determination of the model, this study built a model for the influencing factors of vocational college students’ creativity in the online learning during the COVID-19 based on ECT, TTF, and TAM, and identified seven main constructs. At the same time, it is assumed that gender might adjust each model path. The research model is shown in Figure 1.

### Literature review

#### The expectation confirmation theory (ECM)

Weber (2001) introduced ECM based on Expectation Confirmation Theory (Oliver, 1980). The theory is initially used to study consumers’ purchase and repurchase behaviors, that is, the difference between psychological expectation and actual perception after purchase will affect users’ re-purchase behaviors. In addition, the continuous use of information systems is similar to repeated purchases. As a result, Weber (2001) proposed using ECT to explain users’ intention of continuous use of the information system, borrowing the concept of perceived usefulness from TAM to replace expected use, and modifying ECT to generate ECM.” As research progresses, many academics employ the ECM as the theoretical foundation to explore the continuous use behavior of diverse information systems (Wang et al., 2019; Zuniga-Jara, 2021). For example, Wang et al. (2021b) combined TTF with ECM to test whether online learning assists students complete learning tasks during the pandemic and enhances their willingness to continue using online learning in the future. The results show that the overall research framework largely explains the intention of continuous use. Hai et al. (2020) modified and extended the ECM by adding cognitive and emotional variables (including intrinsic motivation, attitude, and curiosity), with the purpose of exploring the potential factors of continuous learning willingness in a Massive Open Online Course (MOOC) setting. According to the findings, the model could account for 48% of persistent use intentions. Lu et al. (2019) expanded the ECM by adding Flow theory to study the driving factors of MOOC satisfaction and the impact of satisfaction on user behavior. The results show that flow and interest are important variables to improve MOOC satisfaction based on ECT. According to the preceding literature, ECM can be utilized to describe the impact of an online learning environment on students’ actual learning experiences.

#### Technology acceptance model (TAM)

Various theoretical models have been proposed by researchers to explain the factors that influence individuals’ acceptance, rejection, or continued use of new technologies (Ajzen and Fishbein, 1980; Ajzen, 1985; Venkatesh and Michael, 2000; Venkatesh et al., 2003). Based on Ajzen and Fishbie’s Theory of Rational Action (TRA; Davis, 1989) developed TAM to construct a research framework among attitude, intention, and behavior. Two indispensable variables in the model, perceived usefulness and perceived ease of use, will affect the user’s attitude and behavioral intention to use a new technology. TAM is widely used by most researchers in this field, which proves that TAM has significant explanatory power and simplicity, and the theoretical model has become more mature (Tan and Hew, 2017; Esteban-Millat et al., 2018). For example, Wang et al. (2021a) used learning satisfaction theory, TAM, and intelligent learning environments to build a research framework for active online learning, put forward an online intelligent teaching model, and discussed how to promote active online learning in an intelligent environment. Al-Emran et al. (2020) constructed an integrated model that incorporated TAM, planned behavior theory (TPB), and ECM to explain the major predictors of continuous usage of online learning. Al-Adwan et al. (2021) integrate the Information System Success Model (ISSM) and TAM to explain the key factors affecting the success of e-learning systems. Based on the TAM, Wiardi et al. (2022) investigate students’ intentions to reuse online learning systems through IT system quality and perceived usefulness. Alturki and Aldraiweesh (2022) studied students’ satisfaction with mobile learning and their perceptions of the real use of mobile learning during COVID-19 using TAM theory.

#### The task technology fit model (TTF)

With the deepening of research, scholars gradually pay attention to the relationship between information systems and users’ personal behaviors. Goodhue and Thompson studied earlier and proposed a model called TTF. The model assumes that the acceptance of technology depends on how the technology meets the needs of specific tasks and reflects the logical relationship between information technology and task requirements (Goodhuedale and Thompsonronald, 1995). According to this model, in order to ensure the success of any technology, it is necessary to identify the tasks using technology and the fit between tasks and technologies. This task technology fit refers to the consistency between task requirements, personal capabilities, and technical functions. In the context of COVID-19, the online learning model is a technological feature in itself, and learning objectives are a task when the pandemic breaks out. Students’ online learning is expected to be better when the task fits the technology (Wu and Chen, 2016). Many researchers combine TTF with other theories to explain students’ and teachers’ online learning behaviors (Alyoussef, 2021; Kori and Pedaste, 2021). For example, Shanshan and Wenfei (2022) studied the impact of quality factors on the intention of continuous use of MOOCS from the perspective of quality based on teaching and platform, integrating ECM, TTF, flow theory, and trust. Wan et al. (2020) surveyed the determinants of college students’ satisfaction to continue using MOOCs by integrating the unified theory of acceptance and use of technology (UTAUT), TTF, and user satisfaction. Kissi et al. (2018) investigated the students’ desire for video-based instruction (VBI) in the flipped classroom teaching method and expanded the UTAUT model by adding learning family conflict, perceived control over time, and TTF.

### Hypotheses

In the TTF, technical characteristics refer to the technologies used by individuals to perform tasks, and task characteristics refer to the actions taken by individuals when converting input into output. Considering that the fit between task and technology is the core of the TTF model, if the technology can meet the needs of the task, users will be more willing to use the new technology and think that the technology is easy to use. A higher fit indicates a higher perception of ease of use. TTF has an indirect impact on the “perceived usefulness” through the “perceived ease of use” (Yen et al., 2010; Wu and Chen, 2016; Shanshan and Wenfei, 2022). This study integrates TTF into our research framework to investigate its impact on perceived usefulness and perceived ease of use. On this basis, the following assumptions are proposed:

*H1*: The TTF has a significant impact on perceived ease of use of online learning system.

*H2*: The TTF has a significant impact on perceived usefulness of online learning system.

Confirmation is defined as “the user’s perception of the consistency between the information system’s use expectations and its actual performance” (Bhattacherjee, 2001). Bhattacherjee (2001) pointed out that the user’s confirmation of an information system can enhance the user’s perceived usefulness of the platform, and the user’s unacknowledged expectation of the system will reduce the user’s perceived usefulness (Cheng, 2020; Shahzad et al., 2020; Wang et al., 2021b). In addition, when users get the confirmation of technology, their perceived ease of use of technology will become more specific. Therefore, the degree of users’ confirmation of technology will have a positive impact on perceived ease of use (Hong et al., 2006). On this basis, the following assumptions are proposed:

*H3*: The confirmation has a significant impact on perceived ease of use of online learning.

*H4*: The confirmation has a significant impact on perceived usefulness of online learning.

The relationship between perceived ease of use, perceived usefulness, and attitude toward knowledge sharing has been confirmed by existing studies (Gefen et al., 2000; Nguyen et al., 2019). In this study, attitudes refer to students’ subjective feelings, positive or negative, when they use online learning platforms to share knowledge with others. Perceived usefulness refers to vocational college students’ comprehensive judgment of the online learning system to improve their learning effects. Perceived ease of use refers to learners’ judgment on the difficulty of operation and interaction of an online learning system. Davis (1989) pointed out that under the same other conditions, perceived ease of use can predict perceived usefulness, because the increase of perceived ease of use helps to improve performance. Users are more likely to use the system if they can obtain a large amount of more useful information with simple operations while saving time (Gangwar et al., 2015; Arpaci, 2016; Shiau and Chau, 2016). TAM further points out that perceived ease of use is determined by external factors, perceived usefulness is jointly determined by external factors and perceived ease of use, and attitude is jointly determined by perceived usefulness and perceived ease of use. On this basis, the following assumptions are proposed:

*H5*: Perceived ease of use has a significant impact on perceived usefulness.

*H6*: Perceived ease of use has a significant impact on attitudes toward knowledge sharing.

*H7*: Perceived usefulness has a significant impact on knowledge sharing.

In previous studies, different scholars had different views on knowledge sharing. From a learning perspective, Marks et al. (2008) believe that knowledge sharing can help learners study knowledge and improve their abilities—not only pure learning but also the ability to turn knowledge into action. Two key dimensions of perceived ease of use and perceived usefulness in the TAM are used to explain knowledge-sharing behavior (Ahn et al., 2007; Papadopoulos et al., 2013; Ku, 2014). For example, Yu et al. (2010) discovered a significant positive correlation between perceived usefulness and knowledge sharing. When users perceive the usefulness of the online platform and assist themselves, the likelihood of knowledge sharing with others increases. Yuan et al. (2016) demonstrated that perceived usefulness and perceived ease of use have a significant impact on knowledge sharing in online communities. On this basis, the following assumptions are proposed:

*H8*: Perceived ease of use has a significant impact on knowledge sharing in an online learning system.

*H9*: Perceived usefulness has a significant impact on knowledge sharing in an online learning system.

Bukowitz (1999) described knowledge sharing as an activity of knowledge exchange (such as information, professional knowledge, or ability) between people. Knowledge-sharing attitudes can be defined as the degree to which individuals agree with knowledge sharing or actively share knowledge with others (Hutchings and Michailova, 2004). Previous studies have proved that knowledge-sharing attitude is an important predictor of knowledge sharing (Ramayah et al., 2013; Jolaee et al., 2014). Students who have a positive attitude toward knowledge sharing will have a positive impact on their willingness to share, which means that the more positive their attitude toward knowledge sharing, the stronger their willingness to share knowledge with others online (Fauzi et al., 2018). Arpaci (2016) pointed out in his research that knowledge-sharing attitude has a significant impact on knowledge-sharing willingness, and knowledge-sharing willingness has a significant impact on knowledge-sharing behavior. On this basis, the following assumptions are proposed:

*H10*: The attitude of knowledge sharing in online learning has a significant impact on knowledge sharing.

Knowledge sharing can be regarded as a process of information transmission and creativity generation, because it can help learners generate new ideas in the process of communicating with others (Grant, 1996). Previous studies have shown that knowledge sharing contributes to creativity (García-García et al., 2017; Lee, 2018). For example, Koranteng et al. (2019) believed that when students communicate information and knowledge with others, they will increase their creative ideas, so as to improve their creativity. Kirkwood (2010) pointed out that when students interact with others socially and communicate with each other, they build a learning community where they can learn from each other and express their views. Students who participate in this learning community maintain more participation in learning and are more creative in learning. Aamir Amin and Hassan (2011) pointed out that knowledge is the most important factor in promoting individual creativity. We can obtain high-quality information by sharing our knowledge with others. At the same time, we can combine it with our own knowledge, internalize it, and finally generate new ideas. According to (Kirkwood, 2010); Rüzgar (2005), students who investigate more information or knowledge on the Internet are more creative in their learning and can stimulate creativity. On this basis, the following assumptions are proposed:

*H11*: Knowledge sharing has a significant impact on creativity.

## Gender effect

Gender is a fundamental social and cultural construct. Gender differences are taken into account in various studies in the field of organizational behavior (Boateng et al., 2015; Ahanchian et al., 2017; Ganji and Johnson, 2020). More and more researchers are focusing on the differences in technology acceptance between men and women. For example, Tarhini et al. (2014) found in their research that when gender is used as a regulating variable, the explanatory power of the TAM increases to 52%. Ong and Lai (2006) surveyed 67 female and 89 male employees in six different companies in Taiwan. The results showed that women pay more attention to the ease of use of online learning tools, while men pay more attention to perceived usefulness to determine their behavioral intention to use online learning tools. Davis, (1989) pointed out that there are some gender differences in users’ attitudes toward the use of information systems. Although the impact of gender differences on creativity has not been fully clarified, previous studies have suggested that men are more adventurous and curious to try new things (Alfadda and Mahdi, 2021). Therefore, men are more creative than women (Smith et al., 2016). In this context, the relationship between online learning and creativity is expected to be at different levels between male and female groups. If this difference exists, the attitudes of women and men toward knowledge sharing and the impact of knowledge sharing on men’s and women’s creativity are also different. Therefore, it is reasonable to believe that there are gender differences in the impact of online learning on students’ creativity. We can better understand and gain insight into the impact of online learning on students’ creativity by investigating the regulatory role of gender.

## Materials and methods

### Participants

A total of 252 students from four higher vocational colleges in Jiangxi Province participated in the survey. The sampling population was selected from various departments and departments by a random sampling method. Therefore, the extracted sample features can effectively represent the population features. The questionnaire management and collection process must strictly adhere to the survey requirements to ensure effective data collection. In order to ensure the data quality, this study selects the questions according to the time of completing the questionnaire. That is, according to the prediction, the time of completing the questionnaire is about 5–10 min. Therefore, if the completion time is less than 3 min, the data are considered invalid. The statistical results showed that 23 questionnaires are completed in less than 3 min, and 229 valid data remained after elimination. Among them, 131 (64.87%) are males and 98 (35.13%) are females. By grade, 149 (79.49%) are freshmen, 78 (20%) are sophomores, and 2 (0.51%) are juniors. Humanities and social sciences accounted for 168 (61.28%), while natural sciences accounted for 61 (38.72%). In this study, all the participants participated in online learning for more than one semester. Online learning content mainly includes online lectures and discussions, online assignments, and participation in online tests. Descriptive statistics related to the characteristics of the respondents are shown in Table 1. The minimum sample size of the PLS-SEM test follows the tenfold principle, that is, the number of samples is 10 times the number of observed variables of a single potential variable (Barclay et al., 1995). Table 2 shows that the latent variable of the most observed variables is creativity, so the sample size should be greater than 80. Table 1 shows that the sample size is 229, much higher than 80, which meets the requirements.

**Table 1:** Demographic background of study participants (*N* = 229).

Characteristics	Item	Frequency	Percentage
Gender	Male	131	64.87
	Female	98	35.13
Grade	Freshman	149	79.49
	Sophomore	78	20.00
	Junior	2	0.51
Subject Categories	Humanities and Social Sciences	168	61.28
	Natural Science	61	38.72

**Table 2:** Instrument after measurement model assessment.

Construct	Item	Source
Task-technology fit (TTF)	TTF1: Online learning fits with the way I like to learn and study.	Sukendro et al., 2020
	TTF2: Online learning is suitable for helping me complete my academic assignments.	
	TTF3: Online learning is necessary for my academic tasks.	
Confirmation (CON)	CON1: My experience with using the cloud-based e-learning system is better than what I expected.	Bhattacherjee, 2001 Thong et al., 2006 Larsen et al., 2009
	CON2: The service level provided by the cloud-based e-learning system is better than what I expected.	
	CON3: My expectations from using the cloud-based e-learning system are confirmed.	
Attitude toward Knowledge Sharing (ATT)	ATT1: My knowledge sharing with other organizational members is good.	Ajzen and Fishbein, 1980
	ATT2: My knowledge sharing with other organizational members is pleasant.	
	ATT3: My knowledge sharing with other organizational members is valuable.	
	ATT4: My knowledge sharing with other organizational members is wise.	
knowledge sharing(KS)	KS1: E-learning system facilitates the process of knowledge sharing in anytime anywhere settings.	Al-Emran et al., 2018
	KS2: E-learning system supports discussions with my instructor and classmates.	
	KS3: Sharing my knowledge through e-learning system strengthens the relationships with my instructor and classmates.	
	KS4: The e-learning system enables me to share different types of resources with my class instructor and classmates.	
	KS5: The e-learning system facilitates collaboration among the students.	
Perceive ease of use(PEOU)	PEOU1: The e-learning system is easy to use.	Davis, 1989
	PEOU2: Interaction with e-learning system is clear and understandable.	
	PEOU3: The e-learning system is easy for me to manage knowledge.	
	PEOU4: The e-learning system is convenient and user-friendly.	
	PEOU5: The e-learning system is easy to access.	
Individual creativity (IC)	IC1: I regularly come up with creative ideas.	Moulang and Cahan, 2015
	IC2: I regularly experiment with new concepts and ideas.	
	IC3: I regularly carry out tasks in ways that are resourceful.	
	IC4: I often engage in problem-solving in clever, creative ways.	
	IC5: I often search for innovations and potential improvements within my organization.	
	IC6: I often generate and evaluate multiple alternates for novel problems within my organization.	
	IC7: I often generate fresh perspectives on old problems.	
	IC8: When I do not know how to solve a problem, I often make something up on the spot.	
Perceived usefulness(PU)	PU1: Online learning can improve my academic performance.	Bhattacherjee, 2001 Straub and Gefen, 2004
	PU2: Online learning can improve my study effect.	
	PU3: Online learning can improve my study efficiency.	
	PU4: Online learning is very useful to me.	

TTF = task-technology fit, CON = confirmation, PEOU = perceived ease of use, PU = perceived usefulness, ATT = attitude, KS = knowledge share, IC = individual creativity.

### Measurement

The questionnaire consists of two main parts. The first part collects the demographic data of respondents, including age, gender, grade, and subject category. In the second part, the title of the questionnaire is selected from previous studies, which have high statistical reliability and validity. In order to avoid the error caused by single-item measurement in this study, multi-item measurement is used for each potential variable. Each item is assigned by Likert 5-point scale scoring method, in which 1 represents “very disagree,” 2 represents “disagree,” 3 represents “neutral,” 4 represents “agree, “and 5 represents “very agree” (Churchill, 1979). Seven dimensions of the questionnaire are designed according to the core variables and outcome variables of the hypothesis: task-technology fit (three items), perceived usefulness (four items), confirmation (three items), perceived ease of use (five items), knowledge sharing attitude (four items), individual creativity (eight items), and knowledge sharing (five items). There are 32 questions in total. All potential variables meet validity and reliability criteria (see section “Measurement Model”). Table 2 lists all measurement topics and sources.

### Procedure

The research process mainly includes the following steps: Firstly, this study is ethically approved before data collection; Secondly, the link to the complete questionnaire will be sent to the teachers of vocational colleges in China, and then, the teachers will send it to the students of our school to fill in. Finally, before conducting the survey, it is necessary to clearly point out to the respondents: (a) Participants are voluntary,(b) did not receive any training related to creativity before completing the questionnaire, and (c) it is clearly pointed out that all questionnaires are confidential and anonymous and are only used for research to ensure the authenticity of the questionnaire (Matthews, 2017; Fu et al., 2022). The data are collected from April 2 to April 12, 2022.

### Data analysis

In order to analyze the data, this article used Smart PLS 3.0 (PLS) software and carried it out in two stages. The first stage verifies the measurement model to ensure its reliability and validity. The second stage analyzes the structural model and tests the hypothesis path. PlS-SEM is chosen in this study because it has the following advantages: (1) PLS-SEM can test the relationship between multiple potential variables and reduce errors as much as possible (Wang et al., 2013; Chen and Wu, 2017), (2) small sample size (Hair et al., 2016); (3) focus on model prediction ability (Hair et al., 2016); and (4) non-normal distribution of data (Chin et al., 2003). Based on TTF, TAM, and ECM theory, the proposed model consists of six independent variables, one dependent variable, and one control variable, and the model has high complexity. The purpose of this study is to test the predictive power of the hypothesis model. For the above reasons, PLS-SEM is used to analyze the data.

## Results

This study examines the effectiveness and accuracy of the proposed model at two levels: the measurement model and the structural model. The measurement model mainly tests the relationship between the observed variables and the potential variables. The structural model mainly tests the causal relationship between latent variables.

### Measurement model

The measurement model mainly tests the reliability and validity. The purpose of a reliability test is to judge the reliability of the collected data, and Cronbach’s alpha and composite reliability (CR) are used as measurement indicators. In general, Cronbach’s alpha is greater than 0.70, indicating good internal consistency (Nunally, 1978; Kannan and Tan, 2005). Table 3 shows that the Cronbach’s alpha values of the seven potential variables are higher than 0.70, indicating that the scales of each potential variable have good reliability. At the same time, the composition reliability (CR) value is greater than 0.7, indicating that the internal correlation between the questions is high (Gefen et al., 2000; Hildreth, 2012). Table 3 shows that the CR value range is 0891–0. 969, which further shows that the reliability of the scale used in this study is ideal.

**Table 3:** Results of confirmatory factor analysis, validity analysis, and reliability test.

Dimension	Items	loadings	CR	AVE	CA
ATT	ATT1	0.919	0.966	0.876	0.953
	ATT2	0.960			
	ATT3	0.945			
	ATT4	0.920			
CON	CON1	0.947	0.959	0.886	0.935
	CON2	0.930			
	CON3	0.946			
IC	IC1	0.842	0.952	0.713	0.942
	IC2	0.860			
	IC3	0.813			
	IC4	0.884			
	IC5	0.876			
	IC6	0.864			
	IC7	0.802			
	IC8	0.813			
KS	KS1	0.838	0.948	0.783	0.931
	KS2	0.880			
	KS3	0.888			
	KS4	0.907			
	KS5	0.911			
PEOU	PEOU1	0.859	0.939	0.755	0.919
	PEOU2	0.861			
	PEOU3	0.872			
	PEOU4	0.878			
	PEOU5	0.874			
PU	PU1	0.925	0.969	0.887	0.957
	PU2	0.947			
	PU3	0.945			
	PU4	0.949			
TTF	TTF1	0.882	0.891	0.792	0.868
	TTF2	0.926			
	TTF3	0.860			

α, Cronbach’s alpha; CR: composite reliability; AVE: average variance extracted; ATT: Attitude; CON: confirmation; IC: Individual creativity; KS: Knowledge sharing; PU: Perceived usefulness; PEOU: Perceived ease of use; TTF: Task technology fit.

#### Convergent validity

In this study, the convergence validity is evaluated by factor loading and average variation extraction (AVE).

Firstly, factor loading: By reflecting the relationship between the observed variable and the potential variable, the factor loading of each potential variable should be higher than 0.70, and the observed variable of less than 0.4 should be eliminated (Churchill, 1979; Henseler et al., 2009). Table 3 shows that the factor loading of all items in this study ranges from 0.802 to 0.960, which is greater than the critical value of 0.70, indicating that the topic has high reliability.

Secondly, Average Variance Extracted (AVE): It is another criterion for evaluating the validity of convergence. The AVE value of each potential variable should be higher than 0.50, which explains more than 50% of the variance of the item (Leguina, 2015). The AVE values of seven potential variables are 0.713–0.887, all of which are higher than 0.5, which indicated that the measurement model has good convergence validity.

#### Discriminant validity

In this study, two methods are used to test the discriminant validity: the confidence intervals of correlation coefficients of two dimensions and cross loading. First, the bootstrapping method is used to calculate the 95% confidence interval of the correlation coefficient of paired constructs. The test standard is that the confidence interval of the correlation coefficient of two constructs does not include 1. The results in Table 4 show that it meets the requirements.

**Table 4:** Bootstrapping for examining discriminant validity.

	Correlation estimated	Lower bound	Upper bound
ECM→PU	0.364	0.203	0.507
ECM→PEOU	0.467	0.314	0.599
PU→ATT	−0.002	−0.120	0.100
PU→KS	0.118	0.013	0.214
ATT→KS	0.110	−0.043	0.254
KS→IC	0.595	0.462	0.725
PEOU→PU	0.119	0.033	0.208
PEOU→ATT	0.724	0.605	0.824
PEOU→KS	0.695	0.555	0.852
TTF→PU	0.444	0.307	0.581
TTF→PEOU	0.311	0.170	0.456

Secondly, the test standard of cross loading shows that there is a high correlation coefficient between each item and its potential variables, which is higher than the correlation coefficient with other potential variables. This means that the test has high discriminant validity (Chin, 1998b; Grégoire and Fisher, 2006; Götz et al., 2010). Table 5 shows that the measurement model has good discriminant validity.

**Table 5:** PLS-loadings and cross-loadings.

	ATT	CON	IC	KS	PEOU	PU	TTF
ATT1	**0.919**	0.480	0.487	0.641	0.697	0.477	0.495
ATT2	**0.960**	0.452	0.456	0.627	0.694	0.468	0.482
ATT3	**0.945**	0.456	0.466	0.653	0.680	0.498	0.480
ATT4	**0.920**	0.391	0.432	0.589	0.630	0.439	0.430
CON1	0.429	**0.947**	0.609	0.625	0.660	0.780	0.820
CON2	0.478	**0.930**	0.594	0.654	0.719	0.743	0.775
CON3	0.437	**0.946**	0.645	0.637	0.683	0.812	0.805
IC1	0.413	0.553	**0.842**	0.499	0.498	0.542	0.513
IC2	0.392	0.552	**0.860**	0.476	0.467	0.537	0.540
IC3	0.440	0.534	**0.813**	0.484	0.522	0.518	0.523
IC4	0.382	0.561	**0.884**	0.517	0.513	0.565	0.525
IC5	0.402	0.566	**0.876**	0.525	0.473	0.526	0.508
IC6	0.386	0.558	**0.864**	0.494	0.481	0.570	0.497
IC7	0.438	0.493	**0.802**	0.456	0.463	0.484	0.463
IC8	0.469	0.597	**0.813**	0.552	0.574	0.589	0.540
KS1	0.560	0.587	0.487	**0.838**	0.745	0.572	0.609
KS2	0.591	0.551	0.520	**0.880**	0.743	0.550	0.560
KS3	0.579	0.650	0.537	**0.888**	0.758	0.613	0.606
KS4	0.657	0.560	0.554	**0.907**	0.760	0.562	0.609
KS5	0.583	0.653	0.532	**0.911**	0.785	0.616	0.633
PEOU1	0.670	0.571	0.448	0.771	**0.859**	0.530	0.602
PEOU2	0.595	0.621	0.563	0.803	**0.861**	0.605	0.583
PEOU3	0.595	0.672	0.555	0.695	**0.872**	0.674	0.656
PEOU4	0.610	0.716	0.570	0.699	**0.878**	0.667	0.643
PEOU5	0.671	0.591	0.435	0.755	**0.874**	0.553	0.586
PU1	0.491	0.745	0.571	0.605	0.653	**0.925**	0.759
PU2	0.467	0.776	0.597	0.607	0.642	**0.947**	0.773
PU3	0.457	0.774	0.614	0.594	0.627	**0.945**	0.783
PU4	0.481	0.818	0.637	0.670	0.703	**0.949**	0.833
TTF1	0.417	0.799	0.524	0.607	0.646	0.770	**0.882**
TTF2	0.454	0.766	0.560	0.619	0.650	0.780	**0.926**
TTF3	0.482	0.700	0.543	0.594	0.589	0.677	**0.860**

In summary, the reliability and validity (including convergence validity and discriminant validity) of potential variables of this model meet the requirements. The bold figures are the factor loading corresponding to the constructs.

### Structural model

Structural model tests include collinearity between potential variables, R^2^ “coefficient of decision or prediction ability,” Q^2^ “prediction correlation,” and f^2^ “effect size,” and the significance level of model path.

#### Collinearity test

Multi-collinearity refers to the linear correlation between variables, that is, an independent variable can be a linear combination of one or more other independent variables. Before the hypothesis test, multi-collinearity needs to be tested among the factors. A variance inflation factor (VIF) is a measure of the severity of multi-collinearity problems in structural equation models. The ideal VIF value should be less than 5 (Hair et al., 2011; Wu and Tian, 2022). Table 6 shows that there is no multi-collinearity problem in our study.

**Table 6:** Correlation and VIF values.

	ATT	CON	IC	KS	PEOU	PU	TTF
ATT				2.092			
CON					3.602	4.098	
IC							
KS			1.000				
PEOU	1.950			3.046		2.276	
PU	1.950			1.950			
TTF					3.602	3.822	

#### Predictive accuracy

The predictive power of the model is evaluated by combining R^2^ and Q^2^. R^2^ represents the ratio of the variance that can be explained by the model to the total variance. As is known, the PLS-SEM aims to maximize the explained total variance of the latent variables in the inner model (Leguina, 2015). R^2^ ranges from 0 to 1. The closer it is to 1, the stronger the explanatory power of the model (Hair et al., 2019). In measuring the explained variance of a potential variable relative to its total variance, The value of R is 0.670 with a substantial value, 0.333 is medium, and 0.190 is weak (Chin, 1998a). Then, Q^2^ is known as a predictive relevance value and is used for evaluating the predictive fit of the inner model. The accepted Q^2^ must be greater than zero (Hair et al., 2014; Kock, 2018). Table 7 shows that the proposed model has sufficient predictive power.

**Table 7:** Predictive accuracy.

**Construct**	**R** ^**2**^	**Q** ^**2**^
ATT	0.518	0.445
IC	0.351	0.245
KS	0.743	0.574
PEOU	0.557	0.415
PU	0.752	0.66

#### Effect size (f^2^)

The effect size refers to the change in R^2^ value after removing relevant structural variables from the model. In other words, the effect size is used to evaluate the actual effect of the independent variable on the dependent variable (Hair et al., 2014; Hair et al., 2019). The acceptable range of effect size ranges from 0.15–0.20, 0.20–0.35, and more than 0.35, representing, respectively, small, medium, and large effects (Cohen, 1988; Wu and Wang, 2018). Table 8 shows the effect size of the full model ranging from 0 to 0.626.

**Table 8:** Effect size (f^2^).

	f2	Effect size
ATT→KS	0.023	Small
CON→PEOU	0.138	Small
CON→PU	0.132	Small
KS→IC	0.547	Large
PEOU→ATT	0.562	Large
PEOU→KS	0.626	Large
PEOU→PU	0.025	Small
PU→ATT	0.000	Small
PU→KS	0.028	Small
TTF→PEOU	0.061	Small
TTF→PU	0.210	Medium

#### Path analysis results

**Figure 2:**
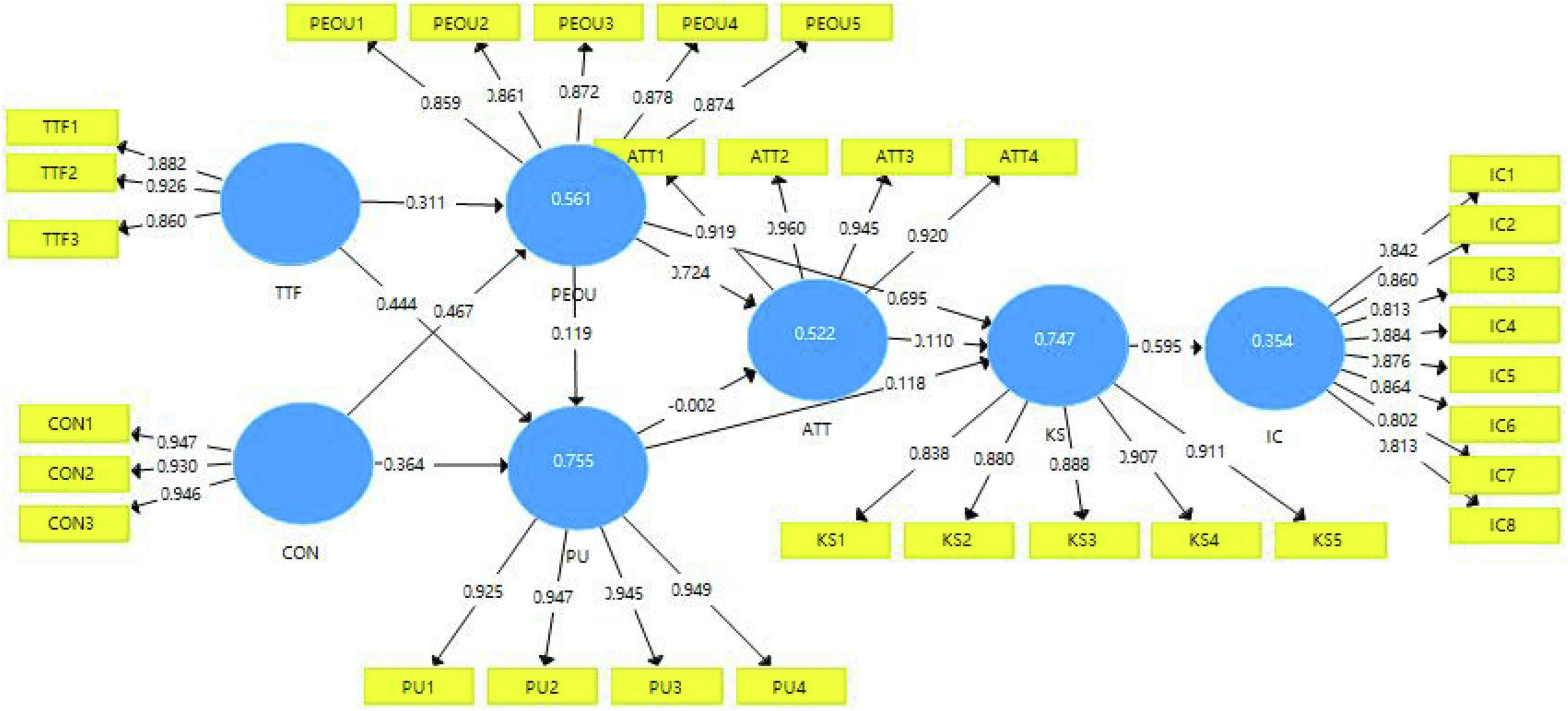
Path coefficients of the research model.

In terms of the hypothesis, the results show that the influence between perceived usefulness and attitude has a weak effect. This is indicated by a value of p of 0.974, so the hypothesis H7 is rejected (*β* = − 0.002, *t* = 0.032, *p* = 0.974). At the same time, ATT does not affect KS (*β* = 0.110, *T* = 1.414, *p* = 0.157) so the hypothesis H10 is rejected. Moreover, other assumptions are supported to varying degrees. Results indicated that the relationships between TTF and PEOU (*β* = 0.311, *t* = 4.185, *p* = 0.000), TTF and PU (*β* = 0.444; *T* = 6.072, *p* = 0.000), CON and PEOU (*β* = 0.467, *t* = 6.988, *p* = 0.000), CON and PU (*β* = 0.364; *T* = 4.700, *p* = 0.000), PEOU and PU(*β* = 0.119; *T* = 2.493, *p* = 0.013), PEOU and ATT (*β* = 0.724; *T* = 11.587, *p* = 0.000), PU and KS (*β* = 0.118; *T* = 2.417, *p* = 0.016), and KS and IC (*β* = 0.595; *T* = 8.842, *p* = 0.000) are all significant. Thus, H1, H2, H3, H4, H5,H6,H8,H9, and H11, as presented in Figure 2, are supported (Table 9).

**Table 9:** Test of hypotheses in the refined model.

	Relationship	value of *t*	Path	value of *p*	Decision
H1	TTF→PEOU	4.185	0.311^***^	0.000	Supported
H2	TTF→PU	6.072	0.444^***^	0.000	Supported
H3	CON→PEOU	6.988	0.467^***^	0.000	Supported
H4	CON→PU	4.700	0.364^***^	0.000	Supported
H5	PEOU→PU	2.493	0.119^*^	0.013	Supported
H6	PEOU→ATT	11.587	0.724^***^	0.000	Supported
H7	PU→ATT	0.032	−0.002	0.974	Not supported
H8	PEOU→KS	9.093	0.695^***^	0.000	Supported
H9	PU→KS	2.417	0.118^*^	0.016	Supported
H10	ATT→KS	1.414	0.110	0.157	Not supported
H11	KS→IC	8.842	0.595^***^	0.000	Supported

* *p* < 0.05; and

*** *p* < 0.001.

## Multi group analysis

Multi-group comparison consists of three steps: first, create a data group; then, test for invariance, and finally, assess group difference.

First of all, create a data group, to assess the differences between the different groups. According to the purpose of the study (Lohmöller, 1989), the number of men and women should not differ greatly. However, if the sample size difference between the two groups is more than 50%, the statistical test results may be biased (Hair, 2015). The number of males and females in this study is 131 and 98, respectively, the difference between the two groups is less than 50%. Therefore, there is no bias in the statistical results.

In the next place, test for invariance. Checking consistency with the MICOM program, Henseler et al. (2015) state that the process consists of three steps: configural invariance, compositional invariance, and equality of composite means value and variances. First of all, the establishment of configuration variance needs to follow the following guidelines: (a) identical indicators per measurement model, (b) identical data treatment, and (c) identical algorithm settings or optimization criteria (Matthews, 2017). In this study, the above criteria are met. Secondly, as suggested by Matthews (2017), the compositional invariance test suggests that original correlations should be equal to or greater than the 5.00% quantile correlations. Table 10 shows that the composite variance test of all potential variables meets the criteria. Third, evaluate the equality of composite variables and mean values in each group. For invariance to be established, the first column (mean original difference) must be a number that falls within the 95% confidence interval. Table 11 shows that the first part of the results does not meet the requirements. The results in Table 12 show that the variance-original difference values of attitude and perceived ease of use do not fall within the 95% confidence interval. Therefore, the second part of the result does not meet the requirements. When steps 1 and 2 are satisfied but step 3 is not, partial measurement invariance is provided. In fact, partial measurement invariance is sufficient to perform PLS-MGA to compare the structural paths between groups, allowing for multi-group analysis (Henseler et al., 2015).

**Table 10:** MICOM step 2 results report.

	Original correlation	5.00%	Permutation values of *p*	Results
ATT	1.000	1.000	0.848	Yes
CON	1.000	1.000	0.833	Yes
IC	0.998	0.998	0.035	Yes
KS	1.000	1.000	0.349	Yes
PEOU	1.000	1.000	0.422	Yes
PU	1.000	1.000	0.033	Yes
TTF	0.999	0.999	0.160	Yes

**Table 11:** MICOM step 3 results report—part 1.

	Mean-original difference (male–female)	Mean-permutation mean difference (male–female)	0.025	0.975	Permutation values of *p*	Equal mean values
ATT	0.478	0.003	−0.263	0.266	0.001	NO
CON	0.389	0.002	−0.255	0.270	0.003	NO
IC	0.331	0.002	−0.262	0.273	0.013	NO
KS	0.478	0.002	−0.259	0.265	0.000	NO
PEOU	0.423	0.003	−0.258	0.270	0.002	NO
PU	0.366	0.002	−0.256	0.261	0.007	NO
TTF	0.475	0.003	−0.256	0.264	0.000	NO

**Table 12:** MICOM step 3 results report—part 2.

	Variance-original difference (male–female)	Variance-permutation mean difference (male–female)	0.025	0.975	Permutation values of *p*	Equal variances
ATT	0.805	0.002	−0.571	0.585	0.007	NO
CON	0.112	0.008	−0.314	0.340	0.504	Yes
IC	0.159	0.006	−0.491	0.519	0.538	YES
KS	0.372	0.003	−0.465	0.497	0.133	YES
PEOU	0.526	0.002	−0.466	0.500	0.034	NO
PU	0.004	0.005	−0.365	0.376	0.982	YES
TTF	0.261	0.005	−0.337	0.358	0.137	YES

**Figure 3:**
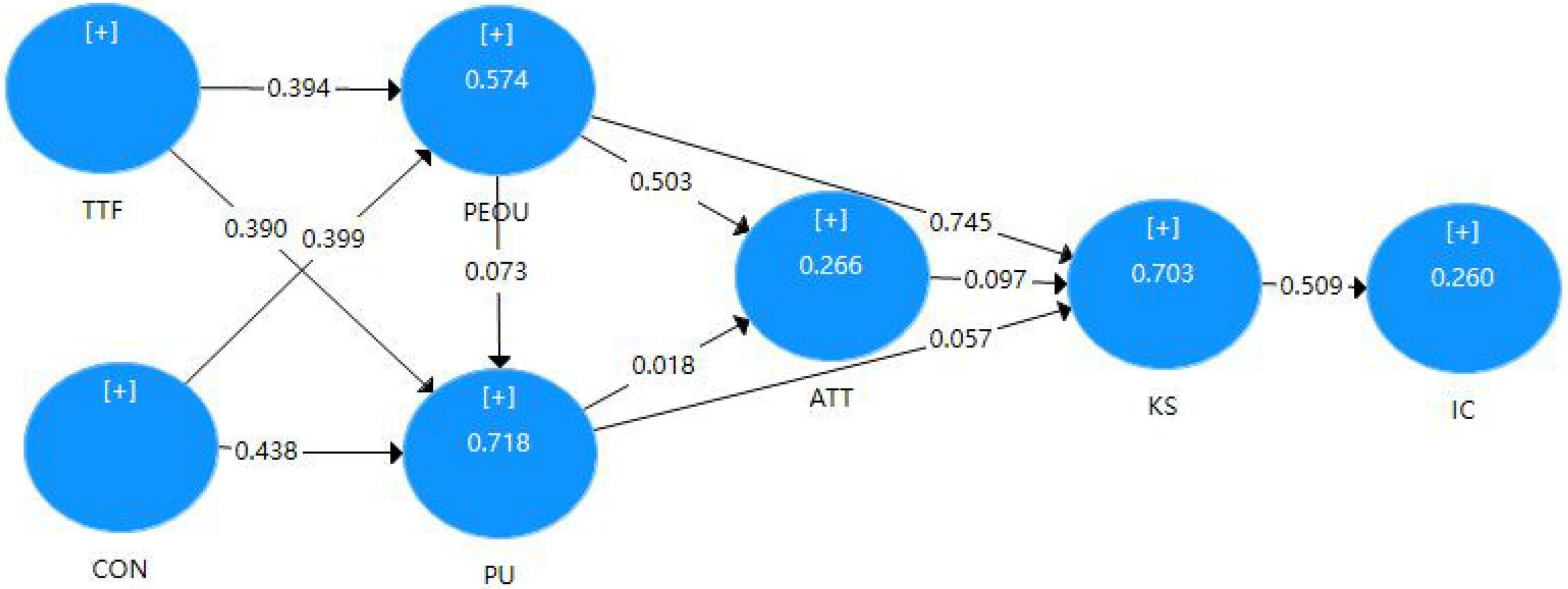
Path model result of male.

**Figure 4:**
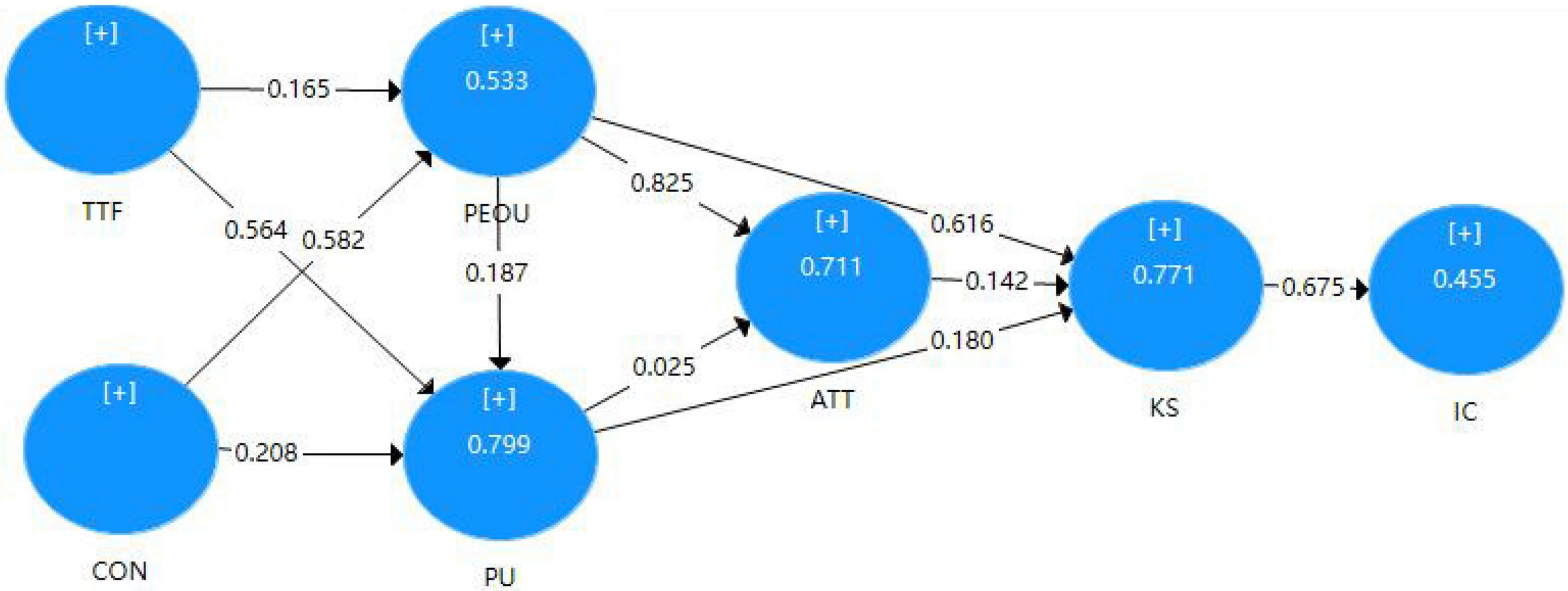
Path model result of female.

Finally, assessment of group differences. After the measurement consistency is established, differences between male and female populations are tested using PLS–MGA. Multi-group analysis is often used to evaluate the differences between the theoretical models proposed by researchers in different sample groups. Table 13 tests the difference between the path coefficient and the composite’s mean. In addition, it is found that there are differences between men and women in their attitudes toward the perceived ease of use of technology and knowledge sharing, while there are no significant differences in other paths (Figures 3, 4).

**Table 13:** Assessment of group differences.

	Path coefficients original (males)	Path coefficients original (males)	Path coefficients original difference (males-females)	Value of *p* original 1-tailed (males vs. females)	Permutation value of *p*	Value of *p* new (males vs. females)	Supported
ATT→KS	0.097	0.142	−0.045	0.620	0.802	0.760	NO
CON→ PEOU	0.399	0.582	−0.182	0.895	0.190	0.210	NO
CON→PU	0.438	0.208	0.231	0.058	0.155	0.116	NO
KS→IC	0.509	0.675	−0.165	0.913	0.247	0.174	NO
PEOU→ATT	0.503	0.825	−0.321	0.996	0.014	0.009	YES
PEOU→KS	0.745	0.616	0.129	0.201	0.419	0.403	NO
PEOU→PU	0.073	0.187	−0.114	0.885	0.245	0.231	NO
PU→ATT	0.018	0.025	−0.007	0.525	0.950	0.950	NO
PU→KS	0.057	0.180	−0.123	0.897	0.236	0.206	NO
TTF→PEOU	0.394	0.165	0.230	0.070	0.136	0.140	NO
TTF→PU	0.390	0.564	−0.175	0.903	0.264	0.194	NO

## Discussion

**Figure 5:**
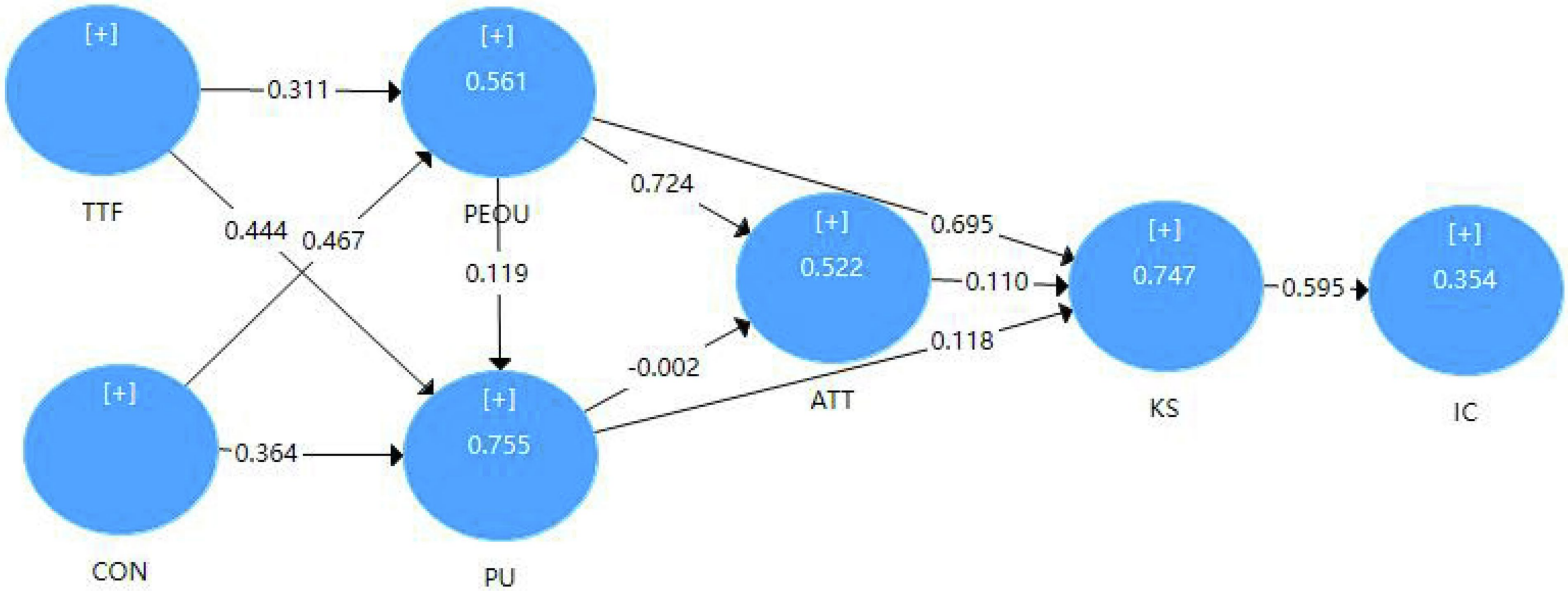
PLS results of the research model.

The purpose of this study is to explore the factors that affect the creativity of vocational college students in online learning situations in the context of COVID-19. The partial least squares method is used to test the research model (Figure 1). The results showed that the majority of the hypothesis relationships between the dimensions of the research model are supported, accounting for 35.4% of the total variance of creativity. The following paragraphs discuss our findings in response to the research questions raised in the introduction. Except for knowledge sharing, all the other dimensions have an indirect influence on creativity. We can see these indirect relationships in Figure 5: TTF → PEOU→PU → KS → IC, TTF → PEOU→PU → KS → IC, PEOU→PU → KS → IC, and ATT → KS → IC.

TTF significantly affects perceived usefulness and TTF significantly affects perceived ease of use, which is consistent with the research results of Pal et al. (2020). Pal and Patra (2021) integrated TAM and TTF models to understand how students view video-based learning during the COVID-19 pandemic. As the epidemic forces learning to be conducted online, only when students believe that using video-based learning can help them achieve their learning goals, they will consider video-based learning to be useful and thus learn effectively. In the context of this study, students are forced to use an online learning system under the influence of the policy. In such an emergency situation, students’ cognition of the usefulness and ease of use of an online learning system will increase, if the online learning system can help them complete learning tasks more efficiently.

The relationship between perceived ease of use and attitude is significantly positive, which is consistent with the research results of Nguyen et al. (2019). They found that perceived ease of use has an indirect impact on employees’ attitudes toward online knowledge sharing through perceived usefulness. However, perceived usefulness is not a strong predictor of knowledge-sharing attitudes in online learning, which is inconsistent with the results of Nguyen et al. (2019). When people do not know how to use a new technology, they tend to focus more on how easy it is to use. But when they know how to use it, they care more about how useful it is (Rahmawati, 2019). The outbreak of the epidemic has forced students to change their traditional learning methods. Therefore, if students think that the online learning platform is easy to use, they will have a sense of achievement, and then use the platform to share knowledge with others will have a positive attitude.

Perceived ease of use significantly affects knowledge sharing, and perceived usefulness significantly affects knowledge sharing. This finding is consistent with Yuan et al. (2016) ‘s prediction that consumers voluntarily share knowledge with others in online communities. Improving vocational college students’ perception of the usefulness of online learning can enhance their behavioral willingness to share knowledge with others in online learning. At the same time, it can also reduce the complexity of online learning platform operations and improve students’ confidence in using online learning systems.

There is no significant correlation between attitudes toward knowledge sharing and knowledge sharing, which is inconsistent with the results of Fauzi et al. (2018). Fauzi et al. (2018) used knowledge sharing as an intermediary to determine the influencing factors of improving academic research productivity, and found that there is a significant positive correlation between attitude toward knowledge sharing and intention of knowledge sharing, and between intention of knowledge sharing and behavior of knowledge sharing. The reason may be that attitude alone may not directly promote behavior, but also needs some external conditions to support it, such as close and frequent interaction between members and fairness of knowledge exchange.

There is a significant positive correlation between knowledge sharing and creativity in the online learning of vocational college students. Lee (2018) applied social technology theory and social capital theory to study the relationship between knowledge sharing and individual creativity. He found that individual creativity can be improved by promoting knowledge sharing among college students. In this study, the more frequent knowledge sharing and the higher the quality of knowledge sharing in the online learning environment of vocational college students, the more students can expand their knowledge boundaries and experience, which is of great value to enhancing students’ creativity.

Confirmation significantly affects perceived usefulness, and confirmation significantly affects perceived ease of use, which is consistent with previous research results (Hong et al., 2006; Thong et al., 2006; Cheng, 2020). Cheng (2020) finds that when users get ERP confirmation experience, their perceived ease of use and perceived usefulness of ERP will become more specific. According to the results, during the COVID-19 outbreak, the online learning platform used to provide services, the quality of the course content, and ease of operation will affect the students to use the experience of online learning platform. If students have a positive experience using the online learning platform, the content of their learning will feel useful and easy to use.

In addition, the moderating effect of gender is examined. The results of the multi-group analysis showed that gender is found to mediate the relationship between perceived ease of use and knowledge-sharing attitudes, and the relationship is stronger for women. This indicates that female students tend to attach more importance to the ease of use of the system when deciding whether to adopt it or not, which is consistent with the findings of Venkatesh and Michael (2000). In this study, there is no significant difference in the influence of gender on other hypothetical relationships in the model. Meanwhile, according to the study by Baer and Kaufman (2011), men are willing to take higher risks and are curious and open to trying new things. As a result, men are more likely to be creative (Kacmar et al., 2011; Smith et al., 2016). However, the findings of this study show that in the online learning situation, the predictive variables of men’s and women’s willingness to use the online learning system are the same, and there is no significant difference in creativity between men and women. The reason for this could be that, in the face of severe acute infectious diseases like covid-19, traditional learning methods have been forced to change, and both men and women have shown great initiative in obtaining useful information through online learning systems, improving academic performance, and stimulating creativity.

Knowledge sharing played a more important role in predicting online learning for women’s creativity than for men’s, as shown in Table 14. This is because, according to the social role theory, men and women have different social expectations, which lead to different social behaviors. Men are more likely to be self-controlling and competitive, whereas women are more likely to cooperate and be willing to socialize with others (Wang et al., 2016). Ma and Yuen (2011) investigated gender differences in knowledge sharing in online learning environments and discovered that women are more likely to share knowledge, whereas men are less likely to share knowledge with others because they are more independent and fear social contact and connection.

**Table 14:** Significance of path coefficient between women and men.

	Value of *p* (female)	Result	Value of *p* (male)	Result
ATT→KS	0.192	Unaccepted	0.337	Unaccepted
CON→PEOU	0.000	Accepted	0.000	Accepted
CON→PU	0.059	Unaccepted	0.000	Accepted
KS→IC	0.000	Accepted	0.000	Accepted
PEOU→ATT	0.000	Accepted	0.000	Accepted
PEOU→KS	0.000	Accepted	0.000	Accepted
PEOU→PU	0.007	Accepted	0.265	Unaccepted
PU→ATT	0.724	Unaccepted	0.847	Unaccepted
PU→KS	0.008	Accepted	0.410	Unaccepted
TTF→PEOU	0.200	Unaccepted	0.000	Accepted
TTF→PU	0.000	Accepted	0.000	Accepted

## Implications

### Practical implications

From the perspective of system developers, with the extensive use of online learning systems in vocational colleges, the results of this study show that the use of online learning systems has a positive impact on cultivating students’ creativity(Kistyanto et al., 2022). Therefore, system developers can further improve the functions of online learning system, customize their own learning plans for each student, and strengthen the communication between students and teachers, so as to further stimulate students’ creative ideas.

From the perspective of teachers, firstly, educators should attach importance to improving students’ creativity, which is critical to improving their ability to solve problems quickly and adapt to social development. Stacey (2007) points out that learning is an interdependent human activity. Our research shows that online learning environments have a positive impact on students’ creativity through knowledge sharing. From this perspective, teachers can encourage students to share knowledge with others in an online learning environment. For example, teachers can help to create a free and open communication environment, encourage students to come up with creative ideas, and give students more freedom and choice in completing tasks. Secondly, teachers should pay attention to the technical characteristics of the online learning system, as prolonged use will result in user fatigue. Therefore, the duration of online courses must be kept within a certain time frame, so that students can continue to participate without becoming bored. Finally, teachers must inform students of the potential benefits of using online learning platforms and how they can help improve their learning outcomes, emphasizing the usefulness of online learning systems to encourage students to use online learning more frequently. The learners’ difficulty in operating the platform is reduced, and their willingness to use the platform is increased, by creating a guide for using the online learning platform and establishing a course assistant to provide Q & A services.

From the perspective of students, the spread of the epidemic has caused anxiety, panic, fear, and a great strain on society, but people’s inherent creativity can turn this difficult time into an opportunity (Fu et al., 2022). Our results show that during the period of the outbreak, schools can cultivate vocational college students’ creativity through online learning because creativity is critical to the ability to quickly solve problems in the high degree of uncertainty created by the pandemic. At the same time, creativity can help students more easily cope with stress and anxiety and can stimulate creative ideas, so as to improve the effect of learning.

### Theoretical implications

This study reveals the impact of online learning on the creativity of students of different genders during the epidemic by integrating TTF, TAM, and ECT models and includes knowledge-sharing variables. An integrated model perspective provides a more complete explanation of the causal mechanism, which cannot be obtained from a single theoretical model. By integrating TTF, TAM, and ECT models and including knowledge-sharing variables, this study reveals the impact of online learning on the creativity of students of different genders during the epidemic. A single theoretical model cannot explain the causal mechanism in its entirety, but an integrated model can. This is a departure from previous studies’ theoretical underpinnings. When compared with earlier research, this is an entirely new approach. Prior research on individual creativity has mostly relied on theories of social and organizational capital.

The results show that the model has 35.1% power to explain the impact of online learning on students’ creativity during the epidemic, indicating that the integration of ECM, TTF, and TAM is theoretically significant. Interestingly, perceived usefulness and attitudes toward knowledge sharing in online learning are not significant. This finding differs from that of Nguyen et al. (2019). Nguyen et al. (2019) integrated TAM and TPB to study the factors influencing employees’ knowledge-sharing behavior in enterprises. The results show that perceived ease of use is significantly positively correlated with employees’ attitudes toward knowledge sharing, and perceived usefulness is significantly positively correlated with attitudes toward knowledge sharing. In addition, perceived ease of use has an indirect impact on online knowledge-sharing attitudes through perceived usefulness. Thus, it adds new controversy to the literature on this topic: Perceived usefulness may not be an important factor when the research objective is knowledge-sharing attitudes in online learning.

In this study, there is no difference in the impact of online learning on creativity of students of different genders. As a result, it has been recognized that in times of extraordinary crisis like the pandemic, both boys and girls can use online learning as a way to stimulate creativity.

## Conclusion

This study combines the TTF, TAM, and ECM models, as well as the knowledge-sharing variable. Simultaneously, it investigates the moderating effect of gender and develops a research model. According to the data analysis results, perceived ease of use and perceived usefulness significantly affect knowledge sharing; knowledge sharing significantly affects creativity. However, there is no significant positive correlation between perceived usefulness and attitude, and there is no significant positive correlation between attitude and knowledge sharing. Furthermore, there are gender differences in the relationship between perceived ease of use and knowledge sharing in online learning; ease of use has a greater impact on women than on men, and online learning has no effect on different students’ creativity. Overall, the proposed model achieved an acceptable degree of fit and explained 35.1% of the variance, revealing that the proposed model can predict and explain the factors that affect students’ creativity in online learning situations to a certain extent.

## Limitations and future directions

Although the results of this study provide new insights, there are still some limitations that may be addressed in future research. First of all, the study population only includes students from a few vocational colleges, resulting in a limited sample range, which may limit the universality of the results. A larger sample size should be used in future studies. Second, this study tests the research model using self-reported data, which may result in deviation. This is because it is impossible to obtain completely objective data for privacy reasons. Therefore, future research should use more objective methods to test the proposed model. Third, the coefficient of determination (R^2^) of the creativity in the model is 35.1%, implying that the total explanatory capacity of all variables is 35.1%. In other words, with the exception of the model in this study, there is a 64.9% chance that other variables can be added to improve the model’s explanatory power.

## Data availability statement

The raw data supporting the conclusions of this article will be made available by the authors, without undue reservation.

## Author contributions

XW: conceptualization and writing—reviewing and editing. XW and XN: data curation. XN: writing original draft. All authors contributed to the article and approved the submitted version.

## Funding

This research was supported by the key base project of Science and Technology Project of Education Department of Jiangxi Province in 2018 (no. GJJ190590); Jiangxi Social Science Planning Project in 2018 (no. 18JY21); Humanities and Social Science Project of Universities in Jiangxi Province in 2020 (no. JY20219); Doctoral Research Foundation of Jiangxi Science and Technology Normal University in 2021 (no. 2021BSQD20); Key Research Base of Humanities and Social Sciences in Universities of Jiangxi Province (no. JD18079); and National Social Science Foundation of China 2017 Education General Project (no. BJA170101).

## Conflict of interest

The authors declare that the research was conducted in the absence of any commercial or financial relationships that could be construed as a potential conflict of interest.

## Publisher’s note

All claims expressed in this article are solely those of the authors and do not necessarily represent those of their affiliated organizations, or those of the publisher, the editors and the reviewers. Any product that may be evaluated in this article, or claim that may be made by its manufacturer, is not guaranteed or endorsed by the publisher.
